# Transcriptional regulators of fetal hemoglobin

**DOI:** 10.1016/j.htct.2024.06.001

**Published:** 2024-08-17

**Authors:** Gabriela Pereira dos Santos, Larissa Teodoro Rabi, André Alves Bezerra, Marcelo Rodrigues da Cunha, Amilton Iatecola, Victor Augusto Ramos Fernandes

**Affiliations:** a Nossa Senhora do Patrocínio University Center, Itú, SP, Brazil; b Laboratory of Cancer Molecular Genetics, School of Medical Sciences (FCM), University of Campinas (UNICAMP), Campinas, SP, Brazil; c Institute of Health Sciences, Paulista University (UNIP), Campinas, SP, Brazil; d College of Medicine of Jundiaí, Jundiaí, SP, Brazil

**Keywords:** Sickle cell anemia, Fetal hemoglobin, Transcription factors, Gene therapy, Gamma-globin

## Abstract

Sickle cell anemia is a hereditary disease caused by sickle-shaped red blood cells that can lead to vaso-occlusive crises. Treatment options are currently limited, highlighting the need to develop new clinical approaches. Studies demonstrated that elevated levels of fetal hemoglobin (Hb F) are associated with a reduction of mortality and morbidity in sickle cell anemia patients. In light of this, researchers have been trying to elucidate the transcriptional regulation of Hb F to develop new therapeutic interventions. The present study aimed to present the main transcription factors of Hb F and discuss the clinical feasibility of these molecular targets. Two search strategies were used in the PubMed, SciELO, and LILACS databases between July and August 2023 to conduct this review. Manual searches were also conducted by checking references of potentially eligible studies. Eligibility criteria consisted of clinical trials and cohort studies from the last five years that investigated transcription factors associated with Hb F. The transcription factors investigated in at least four eligible studies were included in this review. As a result, 56 eligible studies provided data on the BCL11A, LRF, NF-Y, GATA1, KLF1, HRI, ATF4, and MYB factors. The studies demonstrated that Hb F is cooperatively regulated by transcription factors with the BCL11A factor appearing to be the most specific target gene for γ-globin induction. Although these data are promising, there are still significant gaps and intervention limitations due to the adverse functions of the target genes. New studies that clarify the aspects and functionalities of Hb F regulators may enable new clinical approaches for sickle cell anemia patients.

## Introduction

Sickle cell anemia (SCA) is a disease caused by a mutation that leads to abnormal hemoglobin S (Hb S) production. Hb S polymerizes under deoxygenated conditions, causing red blood cells (RBCs) to take on a sickle shape thereby altering their characteristics. Sickled RBCs have a short lifespan and, together with other cell types, can trigger vaso-occlusion of small blood vessels, resulting in multi-organ dysfunction and pain episodes typically occurring in the chest, joints, and limbs. 1, 2, 3

The main therapeutic approaches for SCA involve combinations of palliative medications and preventive measures for symptom management. 1, 2, 3 Although bone marrow transplantation is a curative procedure that suspends sickled RBC production, the limited availability of compatible donors makes this approach restricted and prone to severe complications. Hydroxyurea (HU) is a drug that reduces inflammatory response and improves nitric oxide metabolism, resulting in blood vessel dilation and reduced vaso-occlusion. In addition, HU also inhibits Hb S polymerization by inducing fetal hemoglobin (Hb F) expression. However, the effectiveness of HU is limited due to its inability to induce pancellular conditions and significant Hb F expression in RBCs.^1^, ^2^, ^3^

The Hb F tetramer structure contains two α- and two γ-globin subunits (α²γ²). Thus, Hb F expression depends on γ-globin coding, which occurs through *HBG1* and *HBG2* genes. While the fetal-to-adult hemoglobin switching process represses *HBG1/2* genes, specific mutations can cause hereditary persistence of fetal hemoglobin (HPFH), a condition characterized by persistent γ-globin expression during adulthood. 4, 5, 6

Newborns with SCA and sickle cell patients who co-inherit HPFH exhibit mild symptoms or remain asymptomatic due to Hb F production. The therapeutic benefits of Hb F expression are related to a higher affinity of γ-globin to oxygen and lower Hb S levels. Consequently, Hb S polymerization and the clinical symptoms observed in SCA patients are reduced. 4, 5, 6

Given this, genome-wide association studies (GWAS) have focused on elucidating the regulatory mechanism of γ-globin by identifying transcription factors (TFs) that can be used as molecular targets to induce therapeutic Hb F expression. Several regulatory genes, including *BCL11A, LRF*, and *KLF1*, have been described in the literature. As a result, new treatments for SCA have been developed and recently approved by the Food and Drug Administration (FDA). The interventions are characterized by editing hematopoietic stem cells (HSCs) with CRISPR/Cas9 to increase Hb F production (Casgevy) or via a lentiviral vector to increase hemoglobin A (Hb A) levels (Lyfgenia). 7, 8, 9, 10, 11

In this review, we aimed to identify the main TFs involved in Hb F regulation and explore the clinical application of these molecular targets in SCA treatment.

## Method

### Search strategies

**Figure 1:**
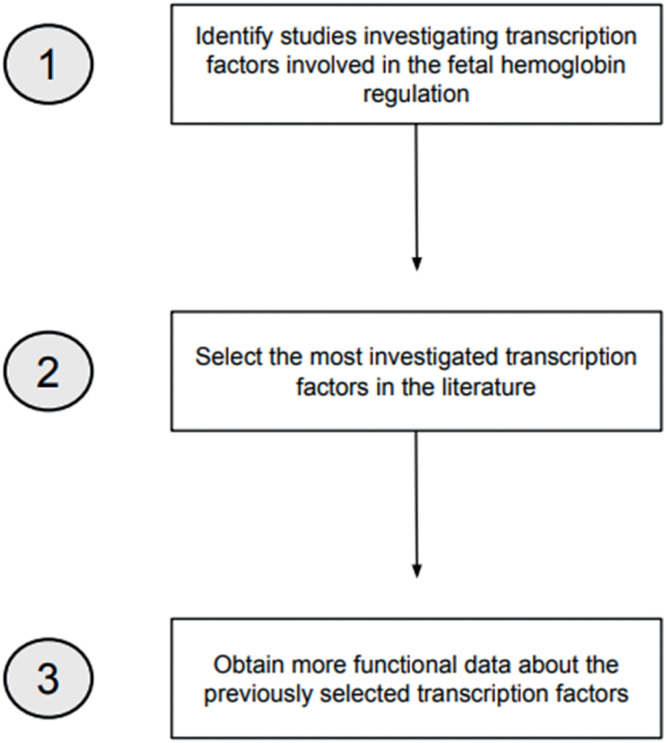
Flowchart demonstrating the search processes applied to identify potentially eligible studies.

TF: transcription factors; SCA: Sickle cell anemia

Two search strategies were conducted in the PubMed, SciELO, and LILACS databases to develop this review. Manual searches were also conducted by checking references of potentially eligible studies. The searches were made in July and August 2023, in English and Portuguese, using the descriptors ‘Fetal Hemoglobin’, ‘Transcription Factors’, and ‘Hemoglobin Regulation’ and the Boolean operators AND and OR. The first search strategy aimed at identifying TFs related to γ-globin regulation within a timeframe of ten years (2013–2023). The second search strategy had two aims: (1) to select the most investigated TFs involved in Hb F regulation and (2) to obtain more functional data about these TFs. To achieve this, all the TFs identified in the first search were searched independently using the terms ‘Fetal Hemoglobin’ and the AND operator (*e.g.* ‘BCL11A’ AND ‘Fetal Hemoglobin’). The timeframe was reduced to five years (2018–2023) to select the most recent data. Figure 1 demonstrates the search strategies employed.

### Eligibility criteria

Experimental gene editing studies and cohort studies analyzing HPFH polymorphisms were eligible to identify the TFs. Studies about regulatory components other than TFs, such as microRNAs (miRNAs), long non-coding RNAs (LncRNAs), and RNA Binding Proteins (RBPs), were not eligible. Additionally, studies that investigate pharmacological agents, physiological components other than TFs, and TFs investigated in less than five eligible studies were also excluded.

### Study selection

**Figure 2:**
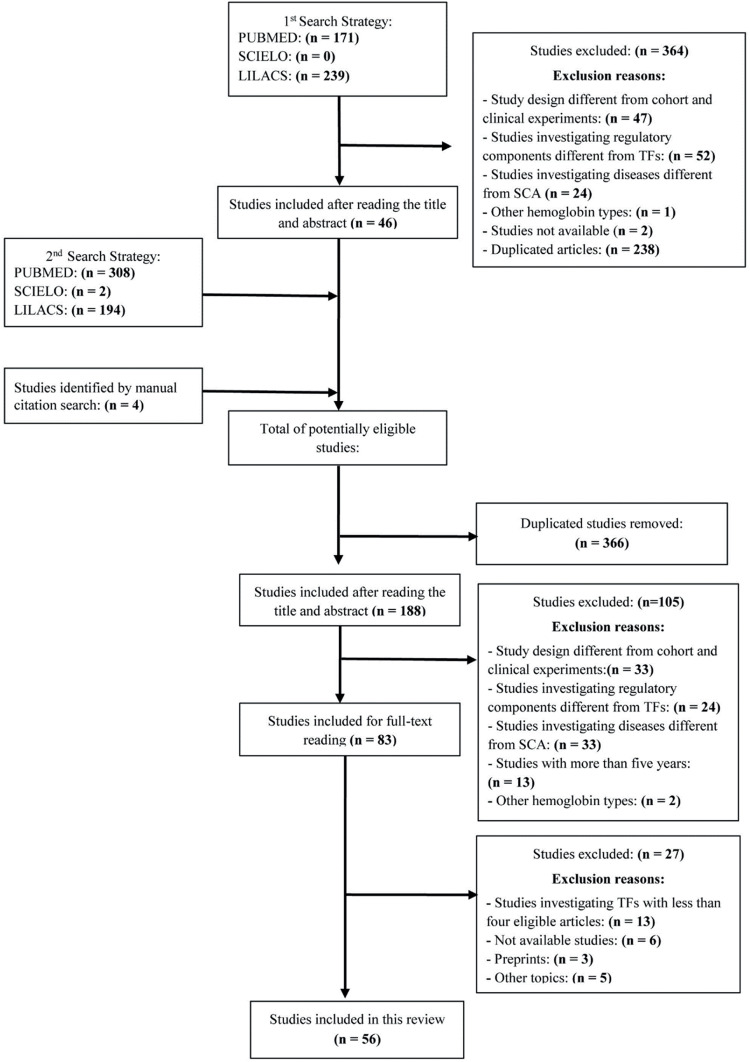
Flowchart demonstrating the identifying, screening, and selecting process of the studies.

The results from the first search were exported to the Rayyan tool and subsequent article selection was based on the title and abstract. After duplicate studies were removed and eligibility criteria applied, each study was labeled with the investigated TF and the respective reasons for inclusion or exclusion. Following this, studies from the second search strategy were exported to Rayyan, and a similar selection and labeling process was performed. Finally, included studies were analyzed by reading the article in full to assess eligibility. The study selection process is presented in the flowchart in Figure 2.

## Results

The search strategies yielded a total of 918 studies, of which 83 were selected for full-text reading, and 56 were eligible to be included in this review. Twenty-seven TFs were identified, of which seven were included in this review as they had been investigated in at least five eligible studies. Table 1 lists the selected TFs. When available, the following data was extracted from eligible studies: TF interaction with Hb F, therapeutic goal, TF physiological mechanism, potential intervention strategies, and editing limitations. The results from data extraction are summarized in Table 2.

**Table 1:** Transcription factors with the number of eligible studies that provided data.

Transcription factor	Eligible studies
BCL11A	41
MYB	14
KLF1	12
LRF/ZBTB7A	9
GATA1	8
ATF4	5
NF-Y	5
ZNF410	3
NFIX	3
NFIA	3
NRF2	2
TAL1	1
SOX6	1
CTCF	1
NonO	1
LDB1	1
ETO2	1
TEAD4	1
KLF4	1
HIC2	1
YY1	0
FOXO3	0
PAX1	0
NF-E2	0
TFCP2	0
RUNX1	0
GATA2	0

Note: Several studies, especially cohort studies, analyze multiple transcription factors simultaneously.

**Table 2:** Transcription factors included in this review.

**transcription factor**	**Hb F Interaction**	**Therapeutic goal**	**Physiological mechanism**	**Possible intervention strategies**	**Limitations**	**References**
*BCL11A*	Directly represses Hb F	Downregulate	- Binds to the HBG1/2 promoters (TGACCA site) - Recruits the NuRD complex - Interacts with NF-Y, GATA1, MYB and/or ATF4	- INDELs in TGACCA motif - INDELs in HB S1L-MYB intergenic region - BCL11A, KLF1 and/or ATF4 cleavage - New binding motifs for NF-Y and GATA1 (HPFH mutations)	Required in brain and B-cell development	^3^^,^^4^^,^^6^^,^^8^^,^^10^, ^11^, ^12^, ^13^, ^14^, ^15^, ^16^, ^17^, ^18^, ^19^, ^20^, ^21^, ^22^, ^23^, ^24^, ^25^, ^26^, ^27^, ^28^, ^29^, ^30^, ^31^, ^32^, ^33^, ^34^, ^35^, ^36^, ^37^, ^38^, ^39^, ^40^, ^41^, ^42^, ^43^, ^44^, ^45^
*MYB*	Indirectly represses Hb F	Downregulate	- Positively regulates BCL11A and LRF - Interacts with ATF4	- INDELs in HB S1L-MYB intergenic region - MYB, ATF4 and/or HRI cleavage	Crucial in hematopoietic cells	^14^^,^^16^, ^17^, ^18^^,^^26^, ^27^, ^28^, ^29^, ^30^^,^^35^^,^^36^^,^^38^^,^^46^^,^^47^
*KLF1*	Indirectly represses and/or directly promotes Hb F production	Downregulate and/or Upregulate	Positively regulates BCL11A and LRF and/or is potentiated by HBG1/2 mutations	KLF1 cleavage or new KLF1 binding motifs (HPFH mutations)	Crucial in erythropoiesis	^9^^,^^11^^,^^16^^,^^17^^,^^21^^,^^29^^,^^30^^,^^40^^,^^42^^,^^48^, ^49^, ^50^
*LRF/ZBTB7A*	Directly represses Hb F	Downregulate	- Recruits the NuRD complex - Regulated by KLF1 and MYB	- KLF1 and/or ZBTB7A cleavage - INDELs in HB S1L-MYB intergenic region	Regulates several physiologic processes	^8^^,^^17^^,^^18^^,^^21^^,^^25^^,^^28^^,^^37^^,^^42^^,^^51^
*GATA1*	Directly and indirectly promotes Hb F expression	Upregulate	- Interacts with BCL11A - Binds to HBG1/2 promoters	New GATA motifs (HPFH mutations)	Crucial in hematopoietic cells	^3^^,^^12^^,^^21^^,^^22^^,^^52^, ^53^, ^54^, ^55^
*ATF4*	Indirectly represses Hb F	Downregulate	- Positively regulated by HRI kinase - Binds to the HB S1L-MYB intergenic region and/or interacts with BCL11A	- HBB, HRI, and/or ATF4 cleavage - INDELs in HB S1L-MYB intergenic region	Crucial in cellular stress response and erythropoiesis	^14^^,^^56^, ^57^, ^58^, ^59^
*NF-Y*	Directly promotes Hb F expression	Upregulate	- Binds to HBG1/2 promoters (CCAAT motif) - Interacts with BCL11A	- New CCAAT motifs - BCL11A cleavage - INDELs in TGACCA motif	Regulates several physiological processes in mammals	^6^^,^^12^^,^^15^^,^^54^^,^^60^

## Discussion

### Locus control region recruits transcriptional interactions

Hemoglobin (Hb) synthesis is a crucial process in erythropoiesis that enables RBCs to bind to oxygen molecules. Hb structure contains four globin subunits transcribed by genes located on chromosomes 11 and 16. On chromosome 11, the *HBB* locus encodes epsilon (ε), gamma (γ), delta (δ), and beta (β) globins. In this context, the locus control region (LCR) interacts with globin genes through chromosomal looping to recruit transcription interactions at different stages of physiological development. ^4^^,^^6^^,^^12^^,^^15^

During the fetal period, Hb needs to bind with a higher affinity to oxygen molecules. Consequently, the LCR interacts with the *HBG1* and *HBG2* genes, leading to γ-globin synthesis and Hb F (α²γ²) expression. The second switching process occurs when the LCR moves from *HBG1/2* to the *HBB* gene, leading to adult hemoglobin (Hb A - α²β²) synthesis through β-globin coding. Although fetal-to-adult hemoglobin switching is a natural and fundamental process in human development, β-globin expression can be problematic. Individuals harboring *HBB* mutations often synthesize abnormal hemoglobins, causing hematological diseases known as hemoglobinopathies. ^1^^,^^2^^,^^4^^,^^6^^,^^12^^,^^14^

### Transcription factors cooperatively regulate fetal hemoglobin

**Figure 3:**
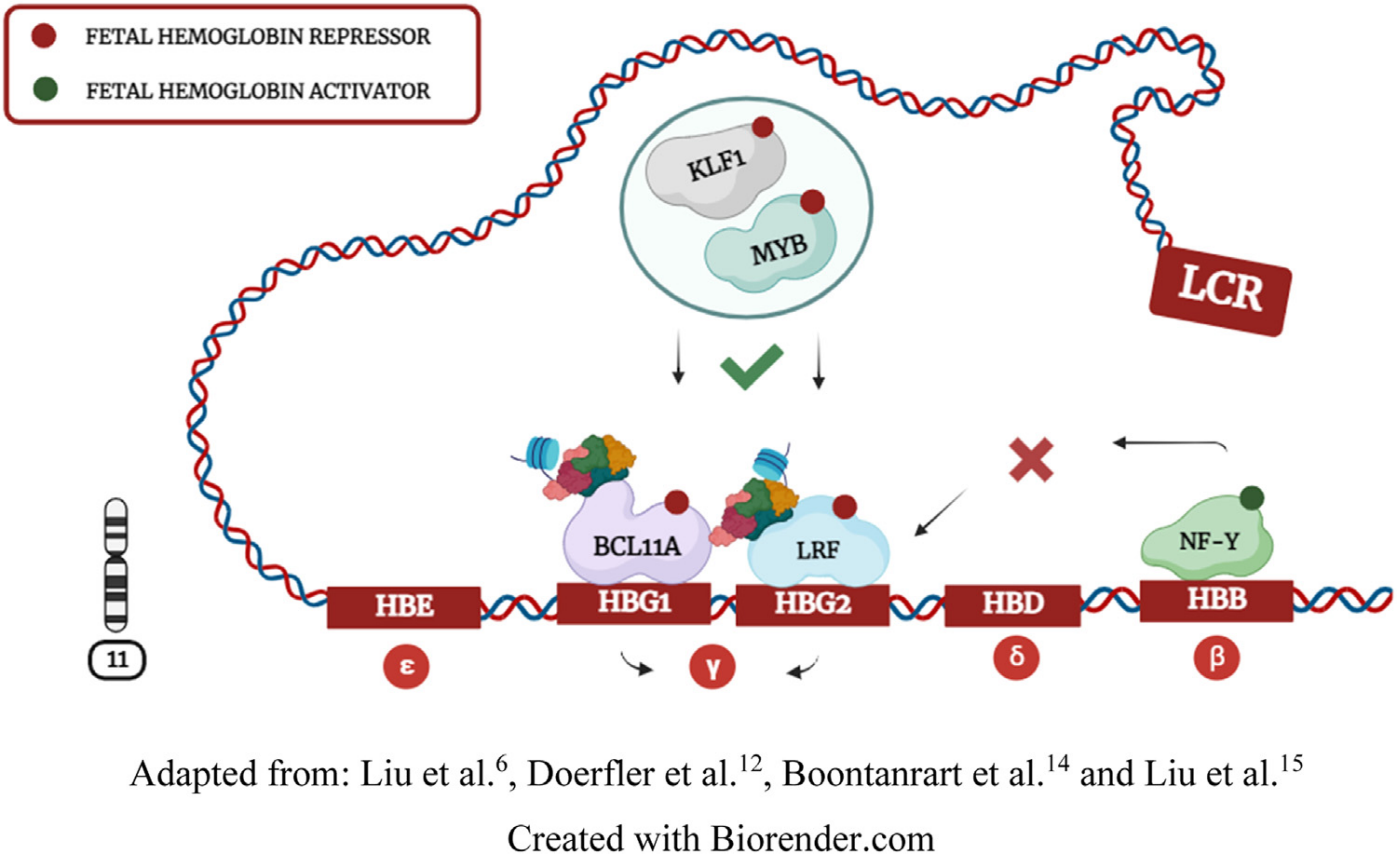
Transcriptional repression of Hb F: illustration demonstrating locus control region (LCR) inducing fetal-to-adult hemoglobin switching through chromosomal looping.

During Hb F repression, *KLF1* and *MYB* factors positively regulate *BCL11A* and *LRF* expression*. BCL11A* and *LRF* repressors bind to *HBG1/2* promoters and recruit the NuRD complex to induce chromatin compaction. Doerfler et al.^12^ demonstrated that compacted chromatin eliminates the *NF-Y* and *GATA1* activators of *HBG1/2* promoters, further contributing to Hb F silencing (Figure 3). LCR interaction with *HBG1/2* genes induces γ-globin expression by inhibiting *BCL11A* and *LRF* repressors and enabling *NF-Y* and *GATA1* activators to directly bind to *HBG1/2* promoters. Some studies suggest that *GATA1* also indirectly regulates γ-globin through *BCL11A* and *KLF1* interactions, but this transcriptional relationship is not yet fully understood. ^4^^,^^6^^,^^11^^,^^14^^,^^15^^,^^52^

**Figure 4:**
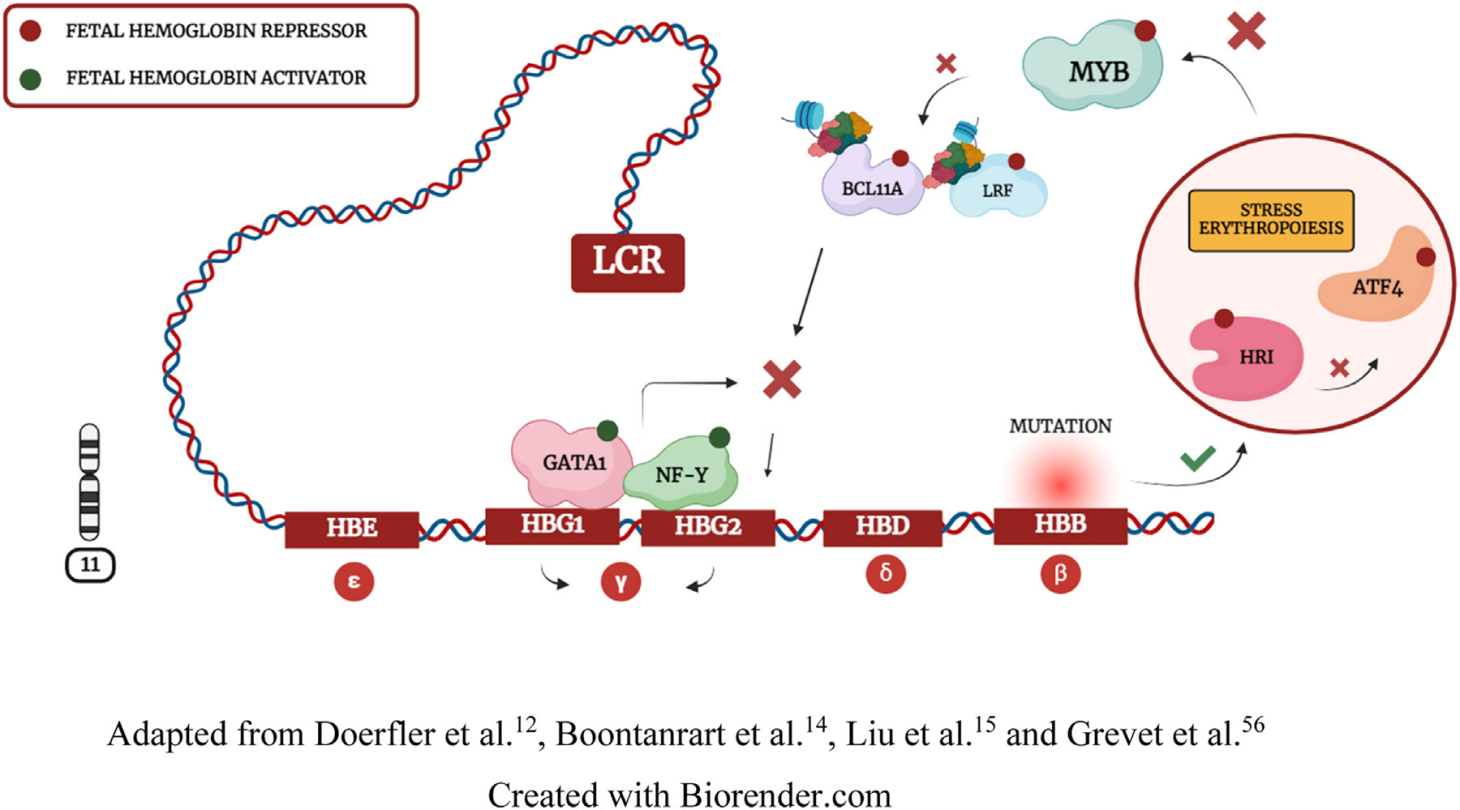
Transcription Activation of Hb F: stress erythropoiesis and a locus control region (LCR) performing chromosomal looping to attract *GATA1* and *NF-Y* activators.

*HRI* is a kinase that phosphorylates eIF2α to regulate protein synthesis. In erythropoiesis, when the integrity of the *HBB* gene is impaired, *HRI* inhibits eIF2α phosphorylation, resulting in *ATF4* repression. Boontanrart et al.^14^ and Huang et al.^57^ demonstrated that *ATF4* downregulation inhibits *BCL11A* expression. Thus, *ATF4* repression leads to *MYB* inhibition and compensatory γ-globin synthesis due to β-globin deficiency (Figure 4). These findings may explain why some HPFH variants occur through mutations in the *HBB* gene and the *HB S1L-MYB* intergenic region. However, while Huang et al.^57^ demonstrated that *ATF4* directly interacts with *BCL11A* to repress Hb F, Boontanrart et al.^14^ demonstrated a specific interaction of *ATF4* with *MYB* to inhibit *BCL11A* and *LRF* repressors. These divergent findings reinforce the need for further investigation regarding *ATF4* molecular interactions. ^14^^,^^17^^,^^56^, ^57^, ^58^, ^59^, ^60^

Collectively, these data demonstrate that Hb F is cooperatively regulated by transcription interactions, and TFs that regulate physiological homeostasis can induce Hb F synthesis when Hb A integrity is impaired. ^4^^,^^6^^,^^12^^,^^14^^,^^17^^,^^52^^,^^56^, ^57^, ^58^, ^59^, ^60^

Although randomized clinical trials are recommended to assess the feasibility of therapeutic interventions, only preclinical studies were identified in the literature. Among the identified studies, only Frangoul et al.^3^ and Esrick et al.^10^ explored the feasibility of interventions in human models. *BCL11A* was the only TF investigated in human experiments, demonstrating that the feasibility of various molecular targets is still uncertain. Despite this, preclinical studies have provided fundamental results that should be further evaluated to guide new clinical interventions.

### *KLF1* is a challenging therapeutic target that may act through divergent mechanisms

*KLF1* is a transcriptional activator of Hb F in −198 T > C, −123 T > C, and −124 T > C mutations. ^7^^,^^22^^,^^43^ However, most studies demonstrated that *KLF1* repression increases Hb F levels, suggesting that *KLF1* may play roles in both repression and activation of γ-globin. ^8^^,^^12^^,^^18^^,^^48^ *KLF1* polymorphisms are associated with the rare blood type In(Lu) and other RBC abnormalities.^11^^,^^50^ Lamsfus-Calle et al.^11^ demonstrated that *KLF1* cleavage can result in dysregulated genes. Moreover, studies demonstrated that *KLF1* haploinsufficient individuals exhibit varied Hb F levels, ^11^^,^^49^ possibly due to other regulatory components associated with *KLF1* functionality and/or a specific mutation inherited by these individuals. Collectively, studies demonstrated that *KLF1* is not yet a safe and effective therapeutic target for Hb F induction. ^11^^,^^50^

### Creating new binding motifs for *NF-Y* and *GATA1* activators

Research has demonstrated that specific HPFH mutations can create new binding motifs for γ-globin activators. ^12^^,^^52^^,^^22^ As a result, researchers have employed gene editing tools to replicate polymorphisms that upregulate *NF-Y* and *GATA1* activators. Because *NF-Y* and *BCL11A* regulate γ-globin through divergent pathways, *BCL11A* editing can promote *NF-Y* binding at the CCAAT motif and result in γ-globin synthesis. Furthermore, the −110 A > C mutation stabilizes *NF-Y* at the CCAAT motif, and the −113 *A* > *G* mutation inhibits the CCAAT motif and creates a new GATA motif to activate Hb F transcription.^12^^,^^52^ Additionally, the disruption of the GATA motif impairs the pancellular distribution of Hb F in RBCs suggesting that *GATA1* may be indispensable for γ-globin synthesis. ^6^^,^^12^^,^^15^^,^^52^

There are still gaps regarding the efficacy and safety of artificial mutations. Initially, for effective inhibition of Hb S polymerization, the artificial polymorphisms must trigger the pancellular HPFH phenotype. 1, 2, 3^,^^52^^,^^53^ Moreover, the use of nucleases to induce double-strand breaks may activate p53-dependent damage responses and lead to the occurrence of large deletions. ^17^^,^^21^^,^^42^ Cheng et al.^17^, Ravi et al.^21^ and Antoniou et al.^42^ demonstrated that base editors may be safer tools for inserting polymorphisms into the genome. The safety of the intervention can also be influenced by transcriptional targets. While *GATA1* dysregulation can cause hematopoietic maturation defects, *NF-Y* disruptions may impair transcriptional events. ^55^^,^^60^ Additionally, Woodard et al.^43^ demonstrated that transgenic mice may not faithfully replicate the human HPFH due to species differences in the genome. Overall, the studies demonstrated that the safety and efficacy of artificial mutations remains uncertain in human models. ^17^^,^^21^^,^^42^^,^^43^^,^^55^^,^^60^

### *MYB* editing may impair efficient hematopoiesis

Several cohort studies have identified polymorphisms in the *HB S1L-MYB* intergenic region. ^14^^,^^16^^,^^28^^,^^37^ As previously mentioned, the safety and efficiency of artificial mutations are still uncertain in human models. ^11^^,^^17^^,^^21^^,^^42^ Although *MYB* cleavage can result in Hb F synthesis, studies evidenced that this TF is crucial for hematopoietic integrity. Clarke et al.^17^ demonstrated that *MYB* haploinsufficiency in mice results in various hematological disorders, suggesting that *MYB* is not a safe molecular target for clinical applications. However, Boontanrart et al.^14^ demonstrated that editing the *HB S1L-MYB* intergenic region results in Hb F expression because it prevents *ATF4* binding, suggesting that *ATF4* may be an efficient molecular target to inhibit *MYB* and activate γ-globin transcription.

### *HRI-*elF2α*-ATF4* pathway integrity is essential to stress erythropoiesis response

*ATF4* repression results in Hb F synthesis by inhibiting *MYB* and *BCL11A*. Because *ATF4* is regulated by the erythroid-specific kinase *HRI,* this approach may offer a more precise way to repress *MYB*. ^14^^,^^56^, ^57^, ^58^, ^59^ However, there are divergences between the studies by Boontanrart et al.^14^ and Huang et al.^57^ regarding the mechanism of action and the effectiveness of *ATF4* as a molecular target. While Huang et al.^57^ demonstrated that *ATF4* interacts directly with *BCL11A* and is not an effective molecular target in mice, Boontanrart et al.^14^ demonstrated that *ATF4* binds to the *HB S1L-MYB* intergenic region and is an effective target in human cells. These findings highlight the need for further studies to elucidate the functionality and effectiveness of *ATF4*.

Because β-globin deficiency results in γ-globin expression due to stress erythropoiesis, artificial *HBB* polymorphisms may also be effective to trigger the *HRI-eIF2α-ATF4* pathway. However, *HBB* deficiency is related to hematological disorders such as β-Thalassemia. Grevet et al.^56^ demonstrated that *HRI* depletion results in Hb F expression, possibly due to subsequent inhibition of *ATF4*. However, Peslak et al.^56^ demonstrated that >80% of *HRI* cleavage is necessary to achieve elevated Hb F levels. Although the study indicated that *HRI* depletion is well-tolerated under homeostatic conditions, it is crucial to assess *HRI* deficiency in pathological conditions, given that this kinase functions primarily during stress events. Zhang et al.^59^ demonstrated that, although healthy mice exhibit normal erythropoiesis, *HRI* depletion under iron deficiency conditions disturbs erythropoiesis. The study also revealed that *ATF4* plays a crucial role in reducing oxidative stress, and *ATF4* depletion is associated with neonatal mouse mortality. These data support the hypothesis that inhibiting homeostatic regulators may be a risky strategy, especially in individuals with hemoglobinopathies.

### *LRF* cleavage can compromise adverse physiological processes

Polymorphisms that disrupt *LRF* binding motifs are associated with Hb F expression. ^7^^,^^17^^,^^18^^,^^25^^,^^42^ Combining *LRF* repression with the recruitment of a transcriptional activator can trigger higher levels of Hb F. These findings have been validated by studies that replicated the −198 T > C mutation, which results in *LRF* repression and creates a new binding motif for *KFL1*. ^7^^,^^21^^,^^42^

*LRF* and *BCL11A* cooperatively regulate Hb F through similar mechanisms. However, the diverse physiological functionality of *LRF* implies that *BCL11A* may be a safer target*.* Ravi et al.^21^ showed that *LRF* cleavage can result in erythroid differentiation defects. Furthermore, Bagchi et al.^3^*^7^* demonstrated that the integrity of erythroid cells remained unaffected after *BCL11A* editing, whereas *LRF* cleavage resulted in erythroid differentiation defects. These data demonstrate that *LRF* is not currently a safe molecular target and that future studies should focus on developing more specific strategies to inhibit *LRF* functionality.

### *BCL11A* is the safest molecular target to induce HB F expression

The *BCL11A* gene encodes a TF especially crucial in the brain, B-cell development and the fetal-to-adult hemoglobin switch. Individuals harboring *BCL11A* mutations can express Dyas-Logan syndrome, a delayed intellectual disorder associated to HPFH. ^27^^,^^45^ Despite the limitations, *BCL11A* is a promising molecular target to induce γ-globin expression due to the possibility of specifically inhibiting this gene function in the fetal-to-adult Hb switching process. ^3^^,^^4^^,^^11^^,^^12^^,^^21^^,^^23^^,^^33^ Insertions or deletions at the TGACCA site of *HBG1/2* promoters or in *BCL11A* erythroid-specific enhancers proved to be highly specific editing strategies for Hb F synthesis. Editing *BCL11A* erythroid-specific enhancers has been applied in rhesus monkeys and human models ^3^^,^^10^^,^^33^, while editing the TGACCA motif has only been employed *in vitro* and in mice. ^4^^,^^6^^,^^11^^,^^12^^,^^21^ Although the sample of clinical experiments is still small, strategically inhibiting *BCL11A* reduces SCA symptoms without affecting other physiologic functionalities of this TF. However, clinical experiments also demonstrated that myeloablative chemotherapy that precedes *ex vivo* interventions is highly toxic for patients. ^3^^,^^11^

*BCL11A* is the exclusive molecular target employed in human experiments due to its effectiveness and safety results in experimental studies. ^3^^,^^4^^,^^6^^,^^10^, ^11^, ^12^^,^^20^^,^^22^^,^^31^^,^^37^ Despite the diverse functionalities exhibited by all the TFs, strategically editing *BCL11A* was the sole intervention that did not affect parallel physiological events. ^3^^,^^9^^,^^11^^,^^32^^,^^37^ The aspects highlighted by studies reinforce that *BCL11A* stands out as the safest and most effective molecular target for Hb F synthesis. Nevertheless, it is crucial to emphasize that implications related to the cerebral and lymphocytic functionality of *BCL11A* underscore the need for meticulous editing analyses to enable clinical applications. ^3^^,^^4^^,^^11^, ^12^, ^13^^,^^22^^,^^24^

### Delivery methods

**Figure 5:**
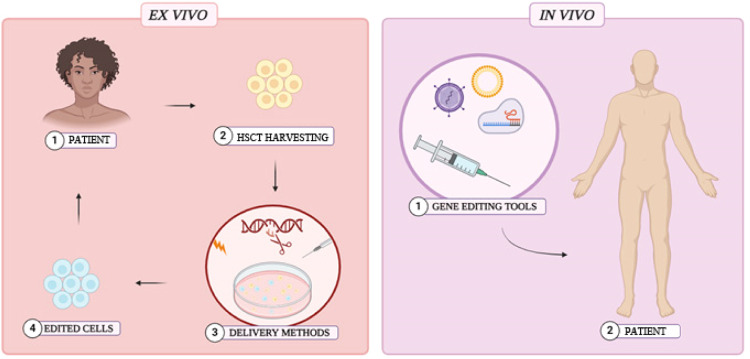
*Ex vivo* and *In vivo* interventions employed for gene editing Created with Biorender.com.

Gene editing is revolutionary for new therapeutic approaches. However, it is still necessary to develop safe and effective methods to deliver editing tools into host cells. Preclinical studies have employed *ex vivo* interventions, where HSCs are extracted, gene-edited, and subsequently reintroduced into the patient after myeloablative chemotherapy. Although *ex vivo* interventions are effective and provide greater control over editing procedures, cell extraction and myeloablation are invasive and potentially toxic. In contrast, *in vivo* interventions offer a safer, more accessible, and simpler alternative, as they eliminate the requirement for myeloablation and HSC harvesting. (Figure 5) ^3^^,^^10^^,^^37^^,^^41^^,^ 61, 62, 63

The feasibility of clinical approaches depends on the strategy applied to introduce editing tools into host cells. Microinjection of the components or the insertion of electrical pulses into cells (electroporation) are highly effective physical methods often employed in genetic interventions. Although Frangoul et al.^3^ demonstrated that electroporation is efficient in human models, it is known that this method can be toxic to HSCs. Furthermore, the viability of physical methods may be restricted to *ex vivo* interventions, making biological methods a promising alternative for *in vivo* implementation. Lentivirus, adenovirus, and adeno-associated viruses (AAV) are commonly employed in biological delivery methods, particularly *in vivo* genetic interventions. The robust infection capacity of these viruses enhances their effectiveness as carriers of genetic tools into target cells, and several studies have underscored the efficiency of viral vectors in genetic interventions. ^9^^,^^37^^,^^41^^,^^61^, ^62^, ^63^ However, each viral method presents specific challenges and advantages. AAV, for example, has a reduced packaging capacity, which can be a challenge to deliver CRISPR/Cas9 ribonucleic complexes into HSCs. Nevertheless, a major concern related to viral vector delivery methods is associated to the potential risks of carcinogenesis and immunogenicity. ^9^^,^^61^, ^62^, ^63^

Multiple studies have explored non-viral biological delivery methods and assessed their safety and efficacy in host cells. 61, 62, 63 Lattanzi et al.^62^ demonstrated that non-viral ribonucleoprotein delivery may be a non-toxic and highly effective delivery method. Similarly, Cruz ^61^ demonstrated that Poly(lactic-co-glycolic acid) (PLGA) nanoparticles exhibit comparable efficiency to viral vectors. However, the study also underscored certain limitations, including the challenge of compounds reaching the bone marrow in *in vivo* interventions. These findings indicate that non-viral biological delivery has the potential to be safer than viral methods, but further efforts are required to optimize the efficiency of non-viral approaches. 61, 62, 63

Finally, it is crucial to empathize that SCA has a high incidence in low-income countries. These aspects underscore the importance of developing cost-effective and simpler approaches. Although most preclinical studies have utilized *ex vivo* interventions with physical or viral delivery methods, it is essential to optimize *in vivo* non-viral delivery to provide more accessible and economical interventions to patients. 1, 2, 3, 4^,^^10^^,^^20^^,^^40^^,^^41^

#### Future perspectives

Several studies have demonstrated that increasing Hb F levels is effective to inhibit the sickling of RBCs. 1, 2, 3, 4, 5, 6^,^^8^^,^^9^^,^^10^ Consequently, TFs that regulate Hb F expression emerge as potential molecular targets to develop new treatments for SCA. Preclinical studies have demonstrated the promising potential of gene therapy in the γ-globin regulation context. 1, 2, 3, 4, 5, 6, 7, 8, 9, 10, 11, 12^,^^14^^,^^20^^,^^21^ Additionally, TFs can also be useful in the exploration and development of new drugs for SCA. Studies have explored the combination of gene editing and pharmacological agents to achieve even higher Hb F levels, demonstrating that γ-globin transcriptional regulation can lead to various therapeutic approaches.^58^ Although more evidence is needed to assess the safety of interventions, the future of new therapies for SCA looks promising. New studies should focus on optimizing current therapeutic strategies to develop safer, effective, and accessible interventions. The evidence resulting from these studies may lead to a broad range of therapeutic options for SCA patients.

## Limitations

This study focuses on discussing the main transcription regulators of Hb F, excluding different regulatory components and those with limited or obsolete data. Gaps and divergences were identified during the literature review. While some studies suggests that *KLF1* is a transcriptional activator of Hb F ^22^^,^^43^^,^^52^, others demonstrated that *KLF1* positively regulates BCL11A and LRF to repress Hb F. ^8,11,18,48^ It is also not completely clear how *KLF1, GATA1* and *MYB* factors interact with each other and other TFs to regulate Hb F. ^13^^,^^15^^,^^54^ In addition, stress erythropoiesis has demonstrated to be an elusive mechanism, as *ATF4* molecular interactions are still not fully understood. ^14^^,^^56^, ^57^, ^58^, ^59^, ^60^ It was also noted that some cohort studies provided limited data, possibly due to the small sample of patients analyzed. ^16^^,^^29^ Finally, it is important to note that transgenic mice have significant differences in the *HBB* locus compared to the human genome. Woodard et al.^43^ demonstrated that this physiological limitation can prejudice the analysis of editing strategies and the understanding of globin regulation.

## Final considerations

The physiological mechanism underlying Hb F expression provides valuable insights to develop new therapeutic approaches for hemoglobinopathies, especially SCA. Although there is a scientific consensus regarding *BCL11A*, there are still implications related to editing limitations and elusive transcriptional interactions. Further studies should focus on elucidating the gaps present in the fetal-to-adult hemoglobin switching process and in the development of new delivery methods to enable accessible and safer interventions for patients.

## Conflicts of interest

The authors declare no conflicts of interest.
